# Supplementary material for: Patterns of Intron Gain and Loss in Fungi
Source: PLoS Biol. 2004 Nov 30;2(12):e422. doi: 10.1371/journal.pbio.0020422 (PMC532390; doi:10.1371/journal.pbio.0020422)
Supplement: Table S1 — Also available at http://genes.mit.edu/NielsenEtAl/. (4.3 MB ZIP). [file pbio.0020422.st001.zip › NielsenEtAl/html/468.html]

AN4883.1.NCU09706.1.MG03854.1.FG06316.1


```
 CLUSTAL W (1.82) Multiple Sequence Alignments - Introns Inserted


Sequence 1: MG03854.1	859 aa
Sequence 2: FG06316.1	881 aa
Sequence 3: NCU09706.1	856 aa
Sequence 4: AN4883.1	816 aa
Alignment Length: 923 aa
Number Identitical Residues: 397 aa
Alignment Score (without introns) 19387


MG03854.1 	MGSPAKKRKLNGDTKPT---PSRGLEYFFAKQKQK----PPEPPLNEYTATSSQV-----
NCU09706.1	MSSPAKKRKLNSGTKQSFSVPSRGLEYFFAKQKQNGASKEPSPLPKESDSQPAQQGSSEE
FG06316.1 	MPSPTKKRKLNNGTKSTA--PAKGLEYFFSKQKQNEAHSSKTPKIEENYGTSSDVPT---
AN4883.1  	MSSPAKKRKRDTQ-------PTRSIASFFQGQTAK------QTEKSEQLASLADTEQ---
          	* **:**** :         *::.:  **  *. :       .  .*  .  ::      

MG03854.1 	-LSDEELARKLQAEWNREDTVRNEAQGVSSKESEGVAARNADGSNLDDATLATDNPLADG
NCU09706.1	HLNDEELARKLQAEWDAEARASSTPREN-DVRSQDVAVSTTEPVDTDG-VEPTNESNPQT
FG06316.1 	EMTDEELARKLQAEWDQE--VANTPHAALQEQTLQLPNEHNVSVSRDESPSPTSTRSKPS
AN4883.1  	HLSDEALARKLQAEWNNE---------------QDFASPATVPHSEPTAASPSPEKDREQ
          	 :.** *********: *                 ..       .   :  .:       

MG03854.1 	SALTVTANSTKTN-----TLTLQSVASAEDTISSVIPLDESPLAFNVSEYITKLKAHWNA
NCU09706.1	VTAPPKADRPKGKN----TLSLSSAAATEDQASASIPLDESPLTFNPSKYIPQLQESWAT
FG06316.1 	ITLPTALNKPSTNNKAKPTLGLQAAGMAEDTTTTSIPFDESPLTFDPAEYVDQLKEHWAS
AN4883.1  	KKAKVG------------VLSLQSSTGTEDTISLSVPFDQSPLVFDSSRYAGELKAHWSA
          	                  .* *.:   :**  :  :*:*:***.*: :.*  :*:  * :

MG03854.1 	EGGNASYAILTRCFVLVSATTSRIKIVDTLVNCLRILIEADPDSLLPA0VWLATNSISPP
NCU09706.1	DGGDASYALLIRCFVLVSSTTSRIKIVDTLVNCVRLLIEGDPSSLLPA~VWLATNAISPP
FG06316.1 	EGGNASYALLTRCFILINGTTSRIKIVDTLVNCLRVLIEGDPASLLPA0VWLATNAISPP
AN4883.1  	EGGNASYALLTRAFVLANSTTSRIKIVDTLVNFLRVLIEGDPSSVLPA~VWLATNSISPP
          	:**:****:* *.*:* ..************* :*:***.** *:*** ******:****

MG03854.1 	~YISLELGLGGSAISKALKQVCGLDSRSLKAIYDKHGDPGDVAFEAKKKQNFTLRKPKPL
NCU09706.1	2--------------------------SLKALNDKLGDPGDVAFEAKKKQSFTLRKPKPL
FG06316.1 	~YISMELGLGGSAISKALRNACGLDNRALKAIYDKHGDAGDVAFEAKKKQSFTLRKPKPL
AN4883.1  	~YHELELGLGGSSISKAFKKIYGLNSQGLKSLYDRLGDAGDVAFEAKKRQSFTLIKPKPL
          	   . . . ..:: :.:  .  . .. .**:: *: **.*********:*.*** *****

MG03854.1 	TIKGVFQSLVKIAQSQGQGSVNVKQQIVDRLLQDARGGEES~RFIVRTLCQH0LRIGAVK
NCU09706.1	TIKGVFQSLVKISKTQGQGSGDAKQRIVDKLLQDARGGEES1----------~-------
FG06316.1 	TIKAVYESLVKIASSQGQGSADTKQRIVDRLLQDARGGEES~RFVVRTLCQH0LRIGAVK
AN4883.1  	SIKGVYQSLIKIAMSKGSGSQELKQRIVEKLLQDTRGAEES~RYIVRTLVQN~LRIGAVK
          	:**.*::**:**: ::*.** : **:**::****:**.***      :  .     .: .

MG03854.1 	TTMLIALSRAFLLSKPYGADFETRDTKELARLKKEELAEVWARGEELVKACFARRPNYND
NCU09706.1	------------------ANIPTKDPNQLAKLKKEELAEVYAKAEEIVKASYARHPDYND
FG06316.1 	TTMLIALSRAFLLSRPPGADFPLKSVTDLAKLKKEDLAEVWGRAEEIVKACFARHPNYND
AN4883.1  	TTMLIALARAFLVSKPDNATFTVHSQHELARLKKEELAEIYSSAEELVKASYARHPNYND
          	::   : : :   : . .* :  :.  :**:****:***::. .**:***.:**:*:***

MG03854.1 	LVPVLLEIGITDELLVRCGLAIHIPLRPMLGSITRDLSEMLTKLQGRDFACEFKYDGQRA
NCU09706.1	LVPVLLETGVSEELLVRCGLTLHIPLRPMLGGITRDLSEMLSRLQGRDFACEYKYDGQRA
FG06316.1 	IVPVLLEIGISEELLLRCGLALHIPLRPMLGSITRDLSEMLTKLQGRDFACEFKYDGQRA
AN4883.1  	LVPCLLEIGVTEELLLRCGLQLHIPLMPMLGSITRDLSEMLTKLQGRDFTCEFKYDGQRA
          	:** *** *:::***:**** :**** ****.*********::******:**:*******

MG03854.1 	QVHCDENGKVSIFSRHLELMTDKYPDLVALIPKIRGEGVSSFILEGEVVAVDSTTGDLKN
NCU09706.1	QIHCDDRGVVSIFSRHLELMTDKYPDLVALVPKIRGEDVQSFIMEGEVVAVDRATGELKN
FG06316.1 	QIHCDENGKVSIFSRHLEVMTDKYPDLVELIPKIRGEGVDSFIMEGEVVAVDRETGELKN
AN4883.1  	QVHCDEAGKVSIFSRHLEEMTEKYPDLVSLVPQIRGEGVSSFILEGEVVAVD-QNGELQP
          	*:***: * ********* **:****** *:*:****.*.***:********  .*:*: 

MG03854.1 	FQTLTNRARKDVEIGSITVNVCLFAFDLMLLNGQPLLNRPFRERRELLKSLFIEVPHNFT
NCU09706.1	FQTLTNRARKDVAIGAITIDVCLFAFDLMYLNGQPLLNRSFRERRDMLRSLFMEIPHHFT
FG06316.1 	FQTLTNRARKDVDIGAITVDVCMFSFDLMHLNGQPLLDRPFRERRELLRSLFKEIPHHFT
AN4883.1  	FQILTNRAKKNVDIGEIKINVCLFAFDLMYLNGAPLLERSLRERRELLRSLFMEIPNRFT
          	** *****:*:* ** *.::**:*:**** *** ***:*.:****::*:*** *:*:.**

MG03854.1 	WVKSIDATSQDSEAVLEFFKAATDQKCEGLMVKILDNLPDIAYKGDG-TTEPVDEQEKIS
NCU09706.1	WVKSIDATSQESEIVLEFFKSAVDSKCEGIMVKILDNLPELEAPGPDDSTDPIIPTELVA
FG06316.1 	WVKSLDATSADSESVLEFFKAAIDIKCEGIMVKILDNLPSALYLGEGEEEEPEGSDRILT
AN4883.1  	WVKSLDATSADSEAVLDFFKSATENKCEGIMVKVLDNT--LATNGSDSTNSSITATP---
          	****:**** :** **:***:* : ****:***:***       * ..  ..        

MG03854.1 	KTTTA-----------KAKGK---------TKTRADN-AEPKRTRRKPLLATYEPDKRLD
NCU09706.1	TSSDAVAVSVSAKKSLKGMGKGNHAETKAPSDTPNPNSNEKKQSRRKPLLATYEPDKRLD
FG06316.1 	PRSKS-----------KGKGK---------SKGKKDETGEKVKTRRKPLLATYEPDKRLD
AN4883.1  	----------------KAEAK-----------------ETKKGGRRKALLSSYEPDKRLE
          	                *. .*                       ***.**::*******:

MG03854.1 	SWLKVKKDYDST---FDTLDLIPIAAWHGQGRKAKWWSPILLAVRNEATGSLEAVTKCMS
NCU09706.1	SWLKVKKDYIGTSAGVDTLDLIPIAAWHGTGRKAKWWSPILLAVRNEETGSLEAVCKCMS
FG06316.1 	SWLKVKKDYNSS---FDTLDLIPVAAWHGQGRKAKWWSPILMACRNEETGSLEAVCKCMS
AN4883.1  	SWLKVKKDYSAS---SETLDLIPVAGWHGNGRKAKWWSPILLAVRNPETGGLQAVTKCMS
          	********* .:    :******:*.*** ***********:* **  **.*:** ****

MG03854.1 	GFTDAFYKENRAFYN-DG--EEDGERQNTRARRPAFVEYSGPEPDVWFEPQEVWEMAFAD
NCU09706.1	GFTDSFYKANREVYDKDGSFSSDGEPKNVHVAKPGFVEYAGGNPDVWFEPQEVWEVAFAD
FG06316.1 	GFTDSFYKANRQFYD-DG--EDGGEPQNTRTTQPSFVEYSGPRPDIWFEPQEVWEMAFAD
AN4883.1  	GFSDKFYQANKEKYE-------EGSPN--VISRPSYVEYNG-EPDVWFEPQEVWEMAFAD
          	**:* **: *:  *:        *. :     :*.:*** * .**:*********:****

MG03854.1 	ITLSPTYTAAIGL~ISDERGLSLRFPRFLKKREDKSIDEASSGEFLATLFRKQEAKAPT-
NCU09706.1	ITISPTYTAAIGL~VREDKGLSLRFPRFLRKREDKGIEEASTSDFLAGLWRKQEVKAPAP
FG06316.1 	ITLSPTYTAAIGL~VSEERGLSLRFPRFMMKRDDKSLEEASTNEFLAGLWRKQEAKAAS-
AN4883.1  	ITLSPTYPAAIGL0RFSDRSLQGISP---MPVDDLQLCYRWQENVKGRILAERASAESIV
          	**:****.*****   .::.*.   *      :*  :      :. . :  ::    .  

MG03854.1 	--VAQG-------IPDDEYE--------
NCU09706.1	PPVEGGREIDEAGLENEDEDDDEYEDGE
FG06316.1 	--KKENSGGAGAQVEDVEEADD------
AN4883.1  	ATQLKRPYVALMNIIDYQT---------
          	..           : : :
```
